# Ranking cancer drivers via betweenness-based outlier detection and random walks

**DOI:** 10.1186/s12859-021-03989-w

**Published:** 2021-02-10

**Authors:** Cesim Erten, Aissa Houdjedj, Hilal Kazan

**Affiliations:** 1 Department of Computer Engineering, Antalya Bilim University, Antalya, Turkey; 2 Electrical and Computer Engineering Graduate Program, Antalya Bilim University, Antalya, Turkey

**Keywords:** Driver gene prioritization, Bipartite graph, Betweenness centrality, Network diffusion

## Abstract

**Background:**

Recent cancer genomic studies have generated detailed molecular data on a large number of cancer patients. A key remaining problem in cancer genomics is the identification of driver genes.

**Results:**

We propose BetweenNet, a computational approach that integrates genomic data with a protein-protein interaction network to identify cancer driver genes. BetweenNet utilizes a measure based on betweenness centrality on patient specific networks to identify the so-called *outlier genes* that correspond to dysregulated genes for each patient. Setting up the relationship between the mutated genes and the outliers through a bipartite graph, it employs a random-walk process on the graph, which provides the final prioritization of the mutated genes. We compare BetweenNet against state-of-the art cancer gene prioritization methods on lung, breast, and pan-cancer datasets.

**Conclusions:**

Our evaluations show that BetweenNet is better at recovering known cancer genes based on multiple reference databases. Additionally, we show that the GO terms and the reference pathways enriched in BetweenNet ranked genes and those that are enriched in known cancer genes overlap significantly when compared to the overlaps achieved by the rankings of the alternative methods.

## Background

Cancer is a complex disease arising in many cases from the effects of multiple genetic changes that give rise to pathway dysregulation through alterations in copy number, DNA methylation, gene expression, and molecular function [1, 2]. Recent cancer genomics projects such as The Cancer Genome Atlas (TCGA) have created a comprehensive catalog of somatic mutations across all major cancer types. A key current challenge in cancer genomics is to distinguish driver mutations that are causal for cancer progression from passenger mutations that do not confer any selective advantage. Consequently, several computational methods have been proposed for the identification of cancer driver genes or driver modules of genes by integrating mutations data with various other types of genetic data [3–10]; see [11–14] for recent comprehensive evaluations and surveys on the topic.

Rather than outputting a set of candidate driver genes or modules, a subclass of cancer driver identification methods output a prioritized list of genes ranked by their cancer driving potential. Early approaches in this group have utilized the mutation frequency of each gene by comparing with background mutation rates [15–17]. However, with a careful review of the existing cancer catalogues it is easy to observe that most tumors share only a small portion of the set of all mutated genes, giving rise to the so called *tumor heterogeneity problem*; methods solely based on mutation rates suffer from low sensitivity due to the existence of long-tail of infrequently mutated genes [4, 18].

One strategy that aims to tackle the long-tail phenomenon is to move from a mutation-centric point of view to a *guilt by association* viewpoint where a correlation between differentially expressed genes and mutated genes are sought. This strategy assumes that even though different sets of genes are mutated in different patients, each of the candidate driver mutations tends to affect a large number of differentially expressed genes. Masica and Karchin present one of the early models based on such a strategy by employing statistical methods for setting up the correlation between mutated genes and the differentially expressed genes to identify candidate drivers [1]. Many different models follow a similar trail by further incorporating biological pathway/network information for setting up such a correlation  [6, 19–23]. DriverNet is among the notable approaches employing mutations data in addition to gene expression and biological network data [19]. It prioritizes mutated genes based on their degrees of network connectivity to dysregulated genes in tumor samples where dysregulation is determined via differential gene expression. Many subsequent approaches are inspired by DriverNet [20–23]. Among them DawnRank [20], the algorithm by Shi et al. [21], and Subdyquency [23] employ, on top of the overall DriverNet model, versions of heat diffusion on the networks integrating data in the form of biological interactions, mutations, and gene expression. Heat diffusion is a technique employed commonly in many cancer driver gene or gene module discovery algorithms [9, 24–28]. It generally serves two purposes simultaneously. On the one hand, since the employed interactions data is usually erroneous, diffusing any type of information through the network of interactions, fixes any potential issues arising from missing links in the network. On the other hand, via the diffusion process, it is possible to observe the extent of an effect such as mutation frequency of a gene, at various distant loci in the network. LNDriver extends the DriverNet concept by taking into account gene lengths of the mutated genes to filter out genes that are mutated with high probability due to their lengths [22]. It should be noted that DawnRank and Subdyquency differ slightly from other approaches; the former can identify patient-specific candidate drivers and the latter employs subcellular localization information in addition to the data made of use in the other methods. There are other driver gene prioritization methods that deviate from the overall guilt by association framework, but nevertheless employ different types of genetic data together with the mutations data. IntDriver utilizes an interaction network and gene ontology data within a matrix factorization framework [29]. Dopazo and Erten employ paired data to generate tumor and normal interaction networks filtered with mutations and gene expression data, and measure the efficacy of various graph-theoretical measures in prioritizing breast cancer genes [6]. Note that among the discussed methods DawnRank also utilizes paired data, both from the tumor and the normal samples.

We propose BetweenNet algorithm for cancer driver gene prioritization. Similar to the methods proposed in [20–23], BetweenNet is also inspired by the DriverNet framework in that it relates the mutated genes and the so-called *outlier genes* corresponding to the dysregulated genes in each patient through a bipartite influence graph. However different from DriverNet and the previous other methods based on it, BetweenNet determines outlier genes based on the betweenness centrality values of the genes in personalized networks. A second contribution of BetweenNet is the employment of a random-walk process on the resulting influence bipartite graph. Random-walks have been utilized in this context previously [21, 23]. However, our application of random walk with restart on the whole influence graph is quite different from the two-step or three-step employment of the diffusion process on a per patient basis described in these methods. Through extensive evaluations we demonstrate that BetweenNet outperforms the alternative methods in recovering known reference genes and in providing functionally coherent rankings when compared to the enriched GO terms or the enriched known functional pathways.

**Fig. 1:**
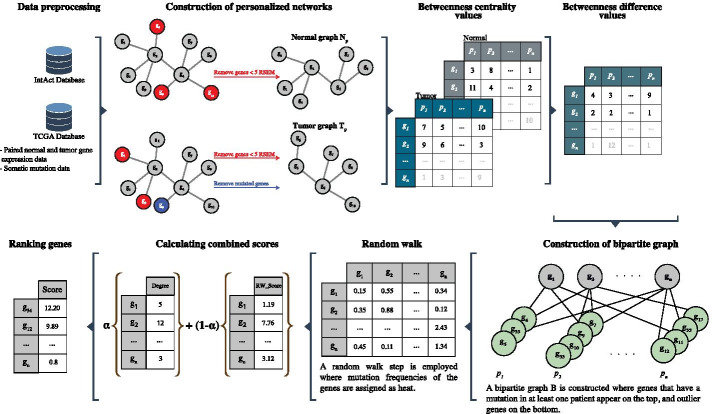
Main steps of the BetweenNet algorithm

**Fig. 2:**
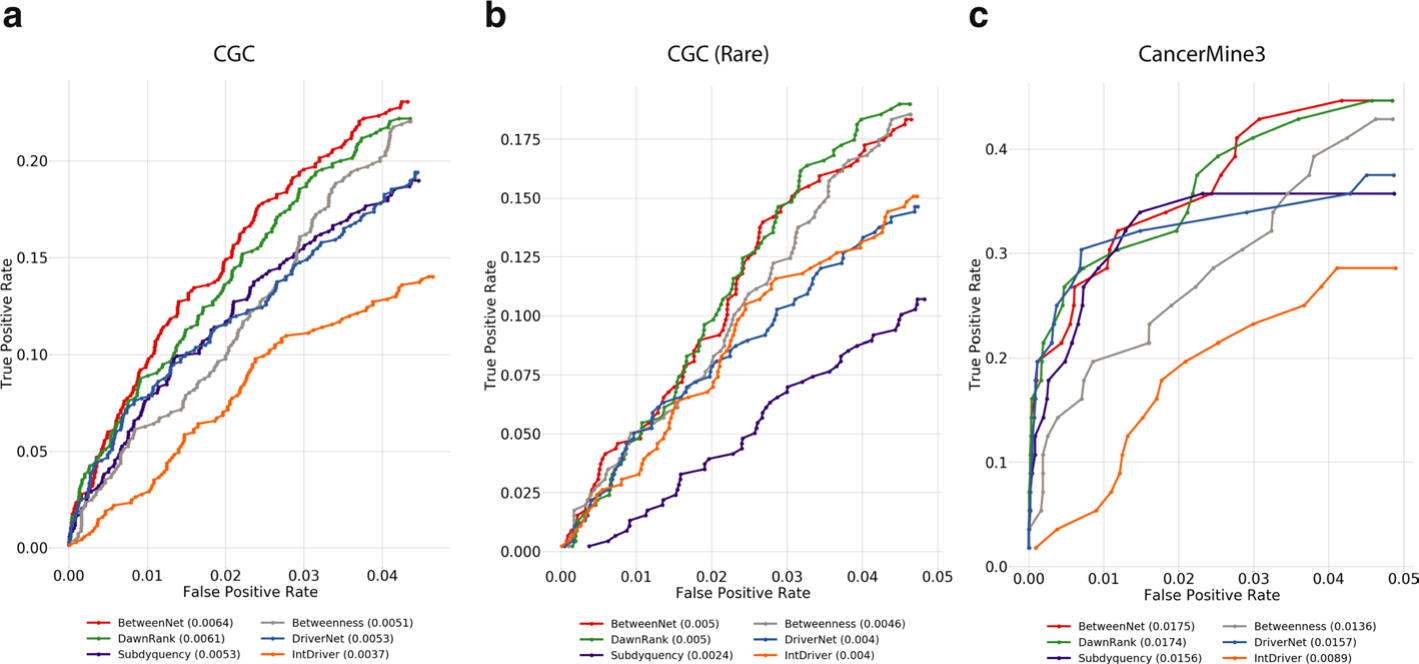
The fraction of recovered reference genes is shown with a ROC curve for lung cancer data **a** *CGC* genes are used as reference. **b** *CGC* rare genes are used as reference. **c** *CancerMine3* genes are used as reference

## Methods

We describe the details of the main steps of the BetweenNet algorithm in this section. Figure 1 provides an overview of the algorithm.

### Input data sets and data preparation

In order to construct the pan-cancer cohort, we first identify the cancer types that have more than 10 paired measurements from normal and tumor samples in the TCGA cohort [30] (Additional file 1: Table 1). We then take the union of all the samples from these cancer types to form the cohort. In addition to the pan-cancer data, we perform separate evaluations on two cancer types. These are breast cancer (BRCA) with 110 samples, lung cancer (LUSC + LUAD) with 61 samples. We download the gene expression (RSEM normalized values [31]) and somatic mutation data for these patients from the Firebrowse database (*http://firebrowse.org*; version 2016_01_28). We exclude the silent mutations in the calculation of mutation frequencies. In addition to the gene expression and mutations data, we also employ protein-protein interactions data which we gather from the *H. Sapiens* PPI network of the IntAct database [32] on 18th June, 2020. We preprocess the IntAct network so that both interactors are proteins and both are from the human genome to avoid human-virus interactions. Also, we only include the interactions where the type is “physical association” or one of its descendants. Next, we convert UniProt ids to gene symbols where we merge multiple UniProt ids for the same protein to a single id. The resulting network contains 15,345 nodes and 113,524 edges.

### Construction of personalized networks

Let $G = (V,E)$ represent the reference *H. Sapiens* PPI network where each vertex $u_i \in V$ denotes a gene *i* whose expression gives rise to the corresponding protein in the network. Each undirected edge $(u_i, u_j) \in E$ denotes the interaction among the proteins corresponding to the genes *i*, *j*. Let *P* represent the set of patient samples. For each patient $p \in P$, we define two graphs $N_p$ and $T_p$ that represent the PPI networks of the normal and tumor samples, respectively. To construct $N_p$, we start with the reference PPI network *G* and remove the nodes that correspond to the genes that are not expressed in the normal sample of the patient *p*. We deem genes with normalized count value less than 5 as not expressed. To construct $T_p$, we remove two sets of genes: (i) genes with normalized count value less than 5 in the tumor sample; (ii) genes that contain non-silent mutations in the tumor sample.

### Calculation of betweenness centrality values

The standard definition of the betweenness centrality ignores the length of a shortest path. Since considering very long paths as functional relations may not be biologically meaningful, we use a variant of the betweenness centrality called *k-betweenness*, where only shortest paths of length $\le k$ are included in the calculations [33]. Given an unweighted graph $G=(V, E)$, k-betweenness value of a node that corresponds to gene *i* is defined as follows:

1 $$\begin{aligned} \sum _{\forall s,t \in V, s\ne i\ne t } \frac{\sigma _{st} (i)}{\sigma _{st}} \end{aligned}$$

where $\sigma _{st}$ is the number of shortest paths of length $\le k$ between genes *s* and *t*, and $\sigma _{st}(i)$ is the number of such paths that pass through gene *i*. We utilize the algorithm presented in Brandes *et al.* to efficiently calculate the k-betweenness values [34]. Let $B^N_{p,i}$ and $B^T_{p,i}$ denote the k-betweenness centrality values of the gene *i* in the $N_p$ and $T_p$ graphs of the patient *p*, respectively. We define $B^{diff}_{p,i}$ as $|B^N_{p,i} - B^T_{p,i}|$.

### Selection of outlier genes

For each gene *i* which exist in both normal and tumor networks, we plot the $B^{diff}_{p, i}$ values across all the patients. We observe that the distribution can be approximated with a truncated normal distribution (Additional file 1: Figure 1). We use the *truncnorm* function in Python to estimate the mean and standard deviation of the distribution. A gene *i* is defined as an *outlier* in patient *p*, if $B^{diff}_{p, i}$ is greater than t standard deviations from the mean. We repeat this process for each gene and construct a set of outlier genes for each patient.

### Construction of the bipartite graph

Similar to DriverNet, we construct a bipartite graph *B* that models the relationship between the set of mutated genes and the outliers. The *mutations partition* of the bipartite graph consists of the genes that have a mutation in at least one patient and the *outliers partition* consists of the outlier genes of all the patients in the cohort. Note that a gene *j* can be an outlier for multiple patients. In such a case, each occurrence of a gene is represented with a distinct node in the outliers partition of *B*. Assuming *j* is an outlier gene for patient *p*, let $u_j^p$ be the node corresponding to it in the outliers partition. For a node $u_i$ in the mutations partition, edge $(u_i,u_j^p)$ is inserted in *B*, if gene *i* is mutated in *p* and $(u_i,u_j)$ is an edge in *G*.

### Random walk on the bipartite graph

We apply a random walk on the bipartite graph *B*. The mutation frequencies of the genes are assigned as initial heat values to be diffused throughout the network during random walk. Let *MF*(*i*) denote the mutation frequency of gene *i*, that is, the number of patients where *i* has a non-silent mutation divided by the total number of patients. Note that heat values are assigned to genes on both sides of the bipartite graph. The random walk starts at a node $u_i$ in *B* and at each time step moves to one of $u_i$’s neighbors with probability $1-\beta$ $(0 \le \beta \le 1).$ The walk can also restart from $u_i$ with probability $\beta$, called the *restart probability*. This process can be defined by a transition matrix *T* which is constructed by setting $T_{ij} = \frac{1}{deg(u_j)}$ if $(u_i, u_j) \in E$, and $T_{ij} = 0$ otherwise. Here, $deg(u_j)$ corresponds to the degree of the node $u_j$. Thus $T_{ij}$ can be interpreted as the probability that a simple random walk will transition from $u_j$ to $u_i$. The random walk process can also be considered as a network propagation process by the equation, $F_{t+1} = (1 - \beta )TF_t + \beta F_0$, where $F_t$ is the distribution of walkers after *t* steps and $F_0$ is the diagonal matrix with initial heat values, that is $F_0[i, i] = MF(i)$. We compute the final distribution of the walk by calculating the *F* matrix iteratively until convergence.

### Ranking genes

Genes in the *mutations partition* of the bipartite graph B are prioritized by a score that combines both degree information and the edge weights that are inferred with random walk. Assuming that $w_{in}(u_i)$ indicates the sum of incoming edge weights for gene *i* after random walk, the combined score for gene *i* can be defined as follows:

2 $$\begin{aligned} S_{i} = \alpha \frac{deg(u_i)}{\max _{\forall u_j \in mutations} deg(u_j)} + (1-\alpha ) \frac{w_{in}(u_i)}{max_{\forall u_j \in mutations} w_{in}(u_j)} \end{aligned}$$

Note that the $w_{in}(u_i)$ for gene *i* corresponds to summing the corresponding row of F for gene *i* after convergence. Once a gene is selected, we remove the corresponding node and its neighbors in *B*. After each such update of the *B* graph, the maximum degree value and the degrees of all the genes are computed again, whereas the $w_{in}$ values are pre-computed and remain fixed throughout the ranking procedure.

### Compiling reference gene sets

We compile known cancer genes from the databases Cancer Gene Census (CGC) [35], Network of Cancer Genes (NCG) [36] and CancerMine [37]. From CGC, we obtain the list of 723 genes that are found to be associated with cancer. We further identify the genes with mutation frequencies $\le 2\%$, namely the *rare drivers*. Since the number of paired samples for breast and lung cancer is small, we use all available samples in breast and lung cohorts to compute the mutation frequencies of genes for defining *rare drivers*. For pan-cancer dataset, the number of paired samples is much larger. Therefore, we calculate mutation frequencies with paired samples only. Furthermore, we filter the genes according to the *Tumour Types* column to define cancer type specific gene sets for breast and lung cancer. We also compile cancer type specific genes from NCG by filtering the *primary site* column. Because these cancer type specific reference gene sets are small we take the union of CGC and NCG cancer type specific reference genes. The third repository, CancerMine, uses text-mining to catalogue cancer associated genes where it also extracts information about the type of the cancer. We compile two lists of genes that have at least 3 and 5 citations, respectively. Hereafter, these two reference gene sets are named *CancerMine3* and *CancerMine5*. The number of genes in each reference set for each cancer type (i.e., lung, breast) and for pan-cancer cohort are available in the Additional file 1: Tables 2-4. For lung cancer, we are unable to use *CancerMine5* as a reference due to its small size.

### Enrichment analysis with gene ontology and pathway databases

For Gene Ontology (GO) [38] term analysis, we use *goatools*. We download go-basic.obo file from http://geneontology.org/docs/download-ontology/ on June 26th of 2019. We restrict the gene annotations to level 5 by ignoring the higher-level annotations and replacing the deeper-level category annotations with their ancestors at the restricted level.

For the pathway analysis, we use the *AllEnricher* tool with Reactome and Kyoto Encyclopedia of Genes and Genomes (KEGG) [39] pathways. Both *goatools* and *AllEnricher* use Fisher’s exact test to calculate p-values and False Discovery Rate (FDR) for multiple testing correction. We use 0.05 as the p-value cutoff to determine significant enrichments.

**Fig. 3:**
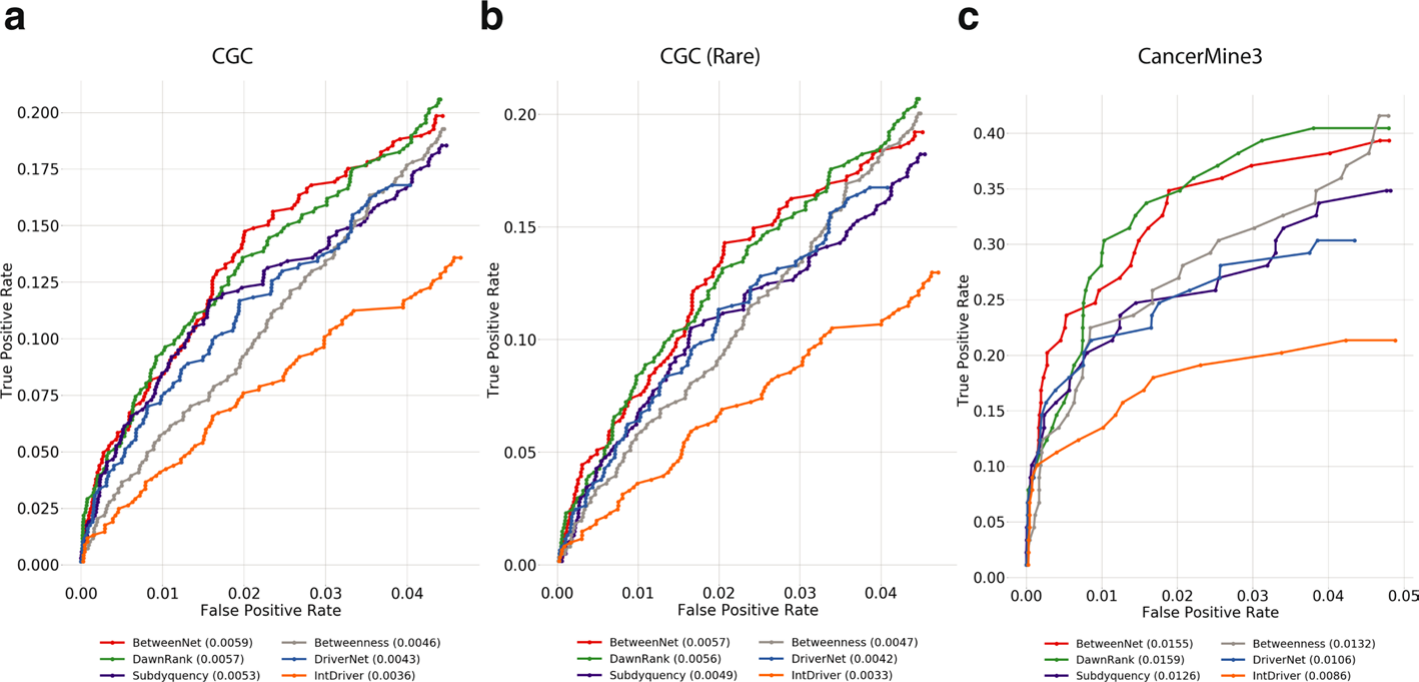
The fraction of recovered reference genes is shown with a ROC curve for breast cancer data **a** *CGC* genes are used as reference. **b** *CGC* rare genes are used as reference. **c** *CancerMine3* genes are used as reference

**Fig. 4:**
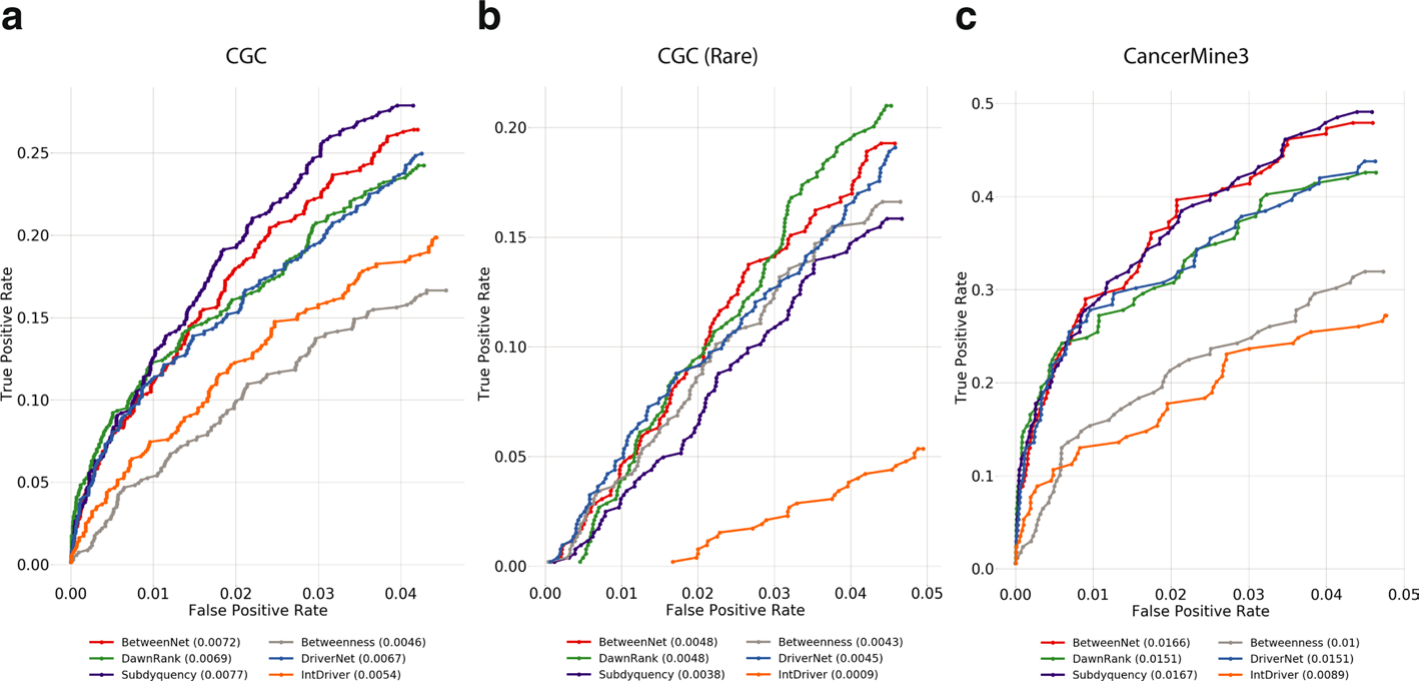
The fraction of recovered reference genes is shown with a ROC plot for pan-cancer data **a** *CGC* genes are used as reference. **b** *CGC* rare genes are used as reference. **c** *CancerMine3* genes are used as reference

## Results

We implemented the betweenness centrality measurement algorithm in C++ using the *LEDA* (Library of Efficient Data types and Algorithms) library. The remaining steps are implemented in Python using *NetworkX* library. All the code and necessary datasets are available at https://github.com/abu-compbio/BetweenNET. We compare BetweenNet results against those of five other existing cancer driver prioritization methods: DriverNet, Subdyquency, DawnRank, IntDriver, and Dopazo and Erten’s prioritization method based on betweenness centrality values, hereafter named only *Betweenness*. Note that for the Betweenness method, although the original method ranks all genes, here we only rank mutated genes using the same method for a fair comparison, since all the other methods under consideration are designed to rank mutated genes only. DriverNet is chosen due to its close connection to our work. DawnRank and Subdyquency are included as they extend and improve over DriverNet. Betweenness is included as a baseline since our method utilizes a variation of betweenness differences in identifying outlier genes. Finally, IntDriver is included to represent the performance of a distinct strategy that is based on matrix factorization. We evaluate the methods with three datasets: lung cancer, breast cancer, and pan-cancer samples.

### Sensitivity of BetweenNet to its parameter settings

We assess the sensitivity of BetweenNet to its parameterization by varying the parameters *t*, $\beta$, $\alpha$ and *k* for lung, breast and pan-cancer samples (Additional file 1: Figures 5 to 18). Among them, the largest change is observed when the outlier detection threshold *t* is increased from 0.5 to larger values. Varying the other parameters results in minimal changes where the changes can only be discerned at the 5th decimal point and beyond. We choose the following setting as it leads to the best performance: $t=0.5$, $\beta =0.4$, $\alpha =0.5$, $k=3$. Overall, these tests show that BetweenNet is robust to a variety of parameter settings.

### Evaluations with respect to reference cancer gene sets

We first compare the methods based on their ability to recover the sets of known cancer genes. For this, we compute true positive and false positive rates for the top 1000 genes and calculate the area under the ROC (AUROC). Figure 2 shows the ROCs obtained from lung cancer data. In Fig. 2a all CGC genes are used as reference, whereas in Fig. 2b genes with mutation frequencies $\le 2\%$, namely the *rare drivers*, are included. Figure 2c is obtained with CancerMine3 as the reference set. BetweenNet achieves a higher AUROC value than all the alternatives for CGC and CancerMine3 reference sets and it has the same AUROC value with DawnRank for CGC-rare reference sets. For CGC and CancerMine3, the ranking of the other methods is the same. Namely, the second ranked method is DawnRank which is followed by Subdyquency and DriverNet with similar performance with respect to each other. Finally, Betweenness and IntDriver are the worst ranking methods. On the other hand, for the CGC-rare reference set, BetweenNet and DawnRank both perform the best whereas the second ranking method is Betweenness. This is followed by DriverNet and IntDriver which have the same AUROC value. Subdyquency performs significantly worse than all the other methods for the CGC-rare reference set. The fact that Betweenness performs much better than DriverNet, Subdyquency and IntDriver is interesting and suggests that most existing methods perform much better in retrieving drivers with larger mutation frequencies. Comparisons using the union of CGC-Lung and NCG-Lung reference sets show that BetweenNet has a significantly better performance than all the other models in retrieving lung cancer specific reference gene sets (Additional file 1: Figure 2). Here, Subdyquency ranks second, which is followed by DawnRank, DriverNet, Betweenness and IntDriver. Overall, these results illustrate the superiority of BetweenNet as it can find both rare and common drivers in lung cancer accurately.

Figure 3 depicts analogous results for the breast cancer data. BetweenNet achieves the top performance with CGC and CGC-rare reference sets. For both reference sets, the ranking of the other methods from best to worst is the same and as follows: DawnRank, Subdyquency, Betweenness, DriverNet and IntDriver. For CancerMine3, DawnRanks shows the best performance. BetweenNet’s AUROC value is slightly worse than DawnRank. This is followed by BetweenNess and Subdyquency. As in the other results of breast cancer, DriverNet and IntDriver are the worst performing methods. Subdyquency ranks the best in retrieving breast cancer specific reference gene sets (union of CGC-Breast and NCG-Breast)(Additional file 1: Figure 3a). BetweenNet’s performance is slightly worse than Subdyquency. The other methods rank as follows: DawnRank, DriverNet, Betweenness, IntDriver. Results with respect to the CancerMine5 reference set are similar to those obtained with CancerMine3 reference set and are available in the Additional file 1: Figure 3b.

Lastly, Fig. 4 shows the results with respect to the pan-cancer dataset. For the CGC reference gene set, Subdyquency performs the best. BetweenNet ranks the second, which is followed by DawnRank, DriverNet and IntDriver, respectively. Interestingly, Betweenness performs the worst in this evaluation. The employed methods rank differently when the reference set is changed to CGC-rare. BetweenNet and DawnRank have a similar performance and perform the best. DriverNet and Betweenness rank the second and third, respectively. Subdyquency ranks the fourth which is surprising given its top performance with the full CGC reference set. For the CancerMine3 reference set, Subdyquency’s AUROC is the highest. BetweenNet’s performance is slightly worse than Subdyquency. DawnRank and DriverNet give the same performance and rank third. Betweenness and IntDriver are the worst performing methods. Results with CancerMine5 are similar to those of CGC and CancerMine3, respectively. These are available in the Additional file 1: Figure 4.

### Evaluations based on functional and pathway analysis

**Fig. 5:**
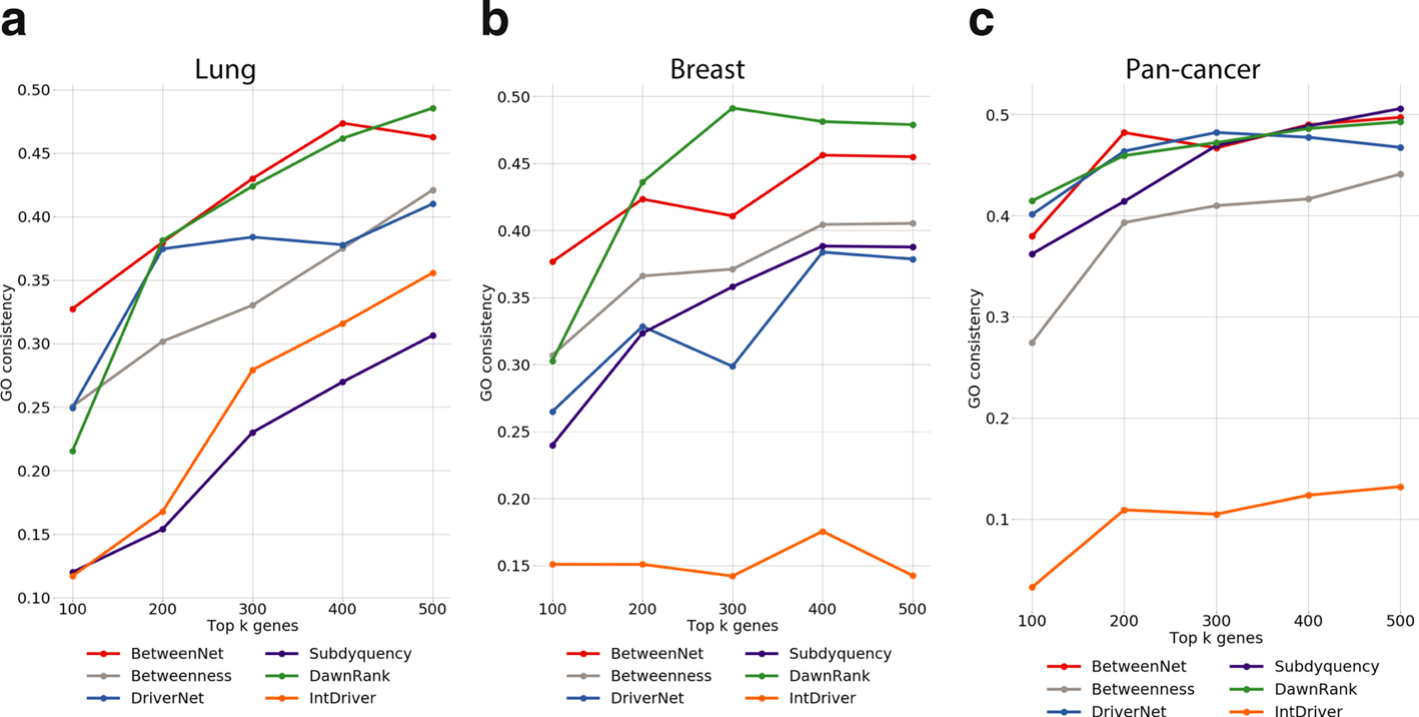
GO consistency values for **a** lung cancer **b** breast cancer **c** pan-cancer cohort

Reference cancer driver gene sets might be incomplete and biased. As such, rather than only finding exact matches between the output gene sets and the reference gene sets, we also define other metrics that measure how well the associated functions of the genes of the two sets match. One such metric is based on GO consistency (GOC) and the other is based on pathway information. For the former, we find the GO terms enriched in the output gene sets and in the reference gene sets, and check whether the corresponding GO terms overlap. The underlying assumption is that the reference cancer genes and the predicted cancer genes should have similar biological functions. We find the enriched GO terms in the ranked gene sets of varying total sizes from 100 to 500 in the increments of 100 for each method under consideration. We repeat the same GO term enrichment analysis with the reference gene set. We then compute the GOC value between the enriched GO terms of the ranked gene set and those of the reference set, which is defined as the ratio between the size of the intersection of the two sets and the size of the union [40]. Figure 5 shows the GOC values calculated for each cancer type and pan-cancer cohort. We observe that BetweenNet ranked genes for lung cancer perform the best for almost all total size values. Here, Subdyquency’s low performance is notable since it performs similar to DriverNet and Betweenness in retrieving CGC genes for lung cancer. For breast cancer, DawnRank ranks the best in four out of five total size values, whereas BetweenNet is the second best. This is followed by Betweenness, Subdyquency and DriverNet, respectively. Finally, IntDriver performs significantly worse than the other methods. For pan-cancer data, there is no clear winner. BetweenNet, DriverNet and DawnRank perform close to each other whereas Subdyquency’s performance is the best for total sizes of 400 and 500. Similar to the results obtained from breast cancer data, IntDriver’s performance is notably worse than all the other methods.

**Fig. 6:**
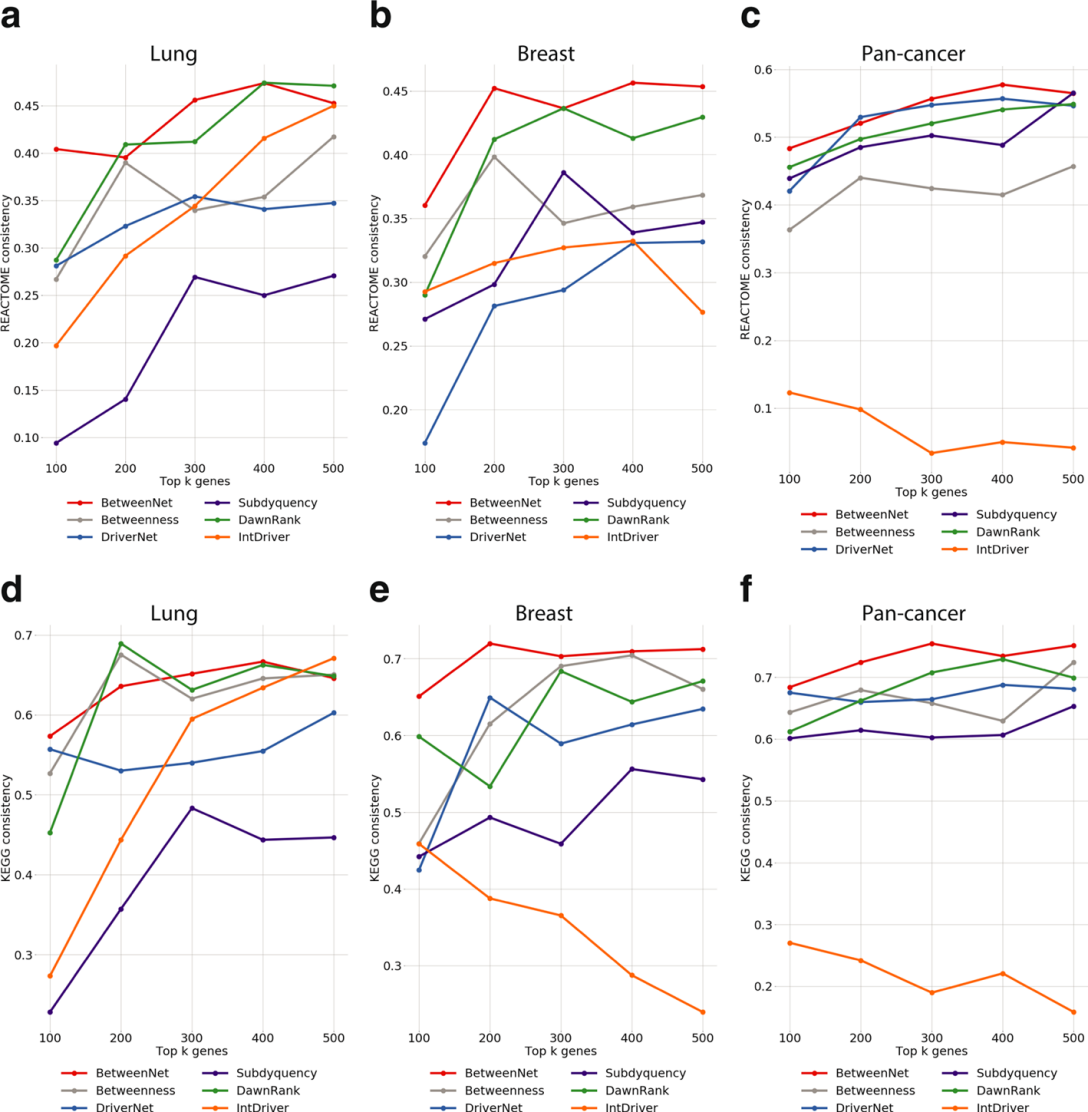
Reactome pathway consistency values for **a** lung cancer **b** breast cancer **c** pan-cancer cohort

We repeat the same type of analysis with pathways as well, this time replacing GO term enrichment with pathway enrichment. Namely, we identify the pathways enriched in the reference set of genes and the set of genes output by a ranking method. We then compute the number of pathways common in both of these sets. Figure 6 shows the results with Reactome reference pathways for all cancer types. For lung cancer, the best method varies for each total size value, where BetweenNet outperforms the other models with a large margin for total sizes 100 and 300. On the other hand, DawnRank results in the top consistency values for total sizes 200 and 400. Finally for total size 400, these two methods share the same performance. For breast cancer, we observe a similar results where BetweenNet and DawnRank perform the top. Here, DriverNet’s performance is notably worse than the other methods. For pan-cancer, BetweenNet gives the top consistency value in four out of five cases. Interestingly, Subdyquency ranks lower than BetweenNet, DawnRank and DriverNet in contrary to its top performance in evaluations with respect to CGC on pan-cancer data. It is less difficult to identify the top performing method when KEGG pathways are used as reference Fig. 6. For lung cancer, BetweenNet gives the best performance for four out five cases, whereas for breast cancer and pan-cancer data BetweenNet ranks top for all total size values. Subdyquency’s low performance is again notable in these evaluations.

## Analysis of BetweenNet ranked genes

We further explore BetweenNet’s top 30 ranking genes for each dataset (Additional file 1: Table 5-7). Among the CGC genes that appear in our top 30 genes for breast cancer, EWSR1 can be found by BetweenNet only whereas ERBB2 and HSP90AB1 can be found by BetweenNet and Betweenness only. We observe that these three genes have lower mutation frequencies than the other CGC genes that appear in our top 30 genes. Namely, HSP90AB1 is mutated in a single patient and the other two genes are mutated in two patients. Similarly, for lung cancer CGC genes SMAD2 and REL can only be detected by BetweenNet and Betweenness within the top 30 ranking genes. Again, these genes have the lowest mutation frequencies among the CGC genes in our top 30 ranking genes. In order for BetweenNet to rank these genes higher than other genes with larger mutation frequencies, many connections must exist between these genes and the outlier sets of the patients that they are mutated in. The fact that these genes cannot be recovered by DriverNet or Subdyquency suggests that defining outlier genes based on betweenness centrality provides an advantage over defining them based on gene expression.

We also check the top 30 ranking genes that do not appear in CGC. Among these, LRRK2 consistently ranks within our top 30 genes for lung cancer, breast cancer and pan-cancer datasets. LRRK2 is also ranked within the top 30 genes by DawnRank, DriverNet and Betweenness for breast cancer dataset; and by all the other methods except IntDriver for lung cancer and pan-cancer datasets. Indeed, multiple studies have reported that individuals with LRRK2 mutations have an increased risk of developing cancers [41–43]. Another gene which is ranked among our top 30 genes for all three datasets is RIF1. RIF1 is also identified by DriverNet and DawnRank in all three datasets. Supporting this finding, RIF1 is recently shown to promote tumor growth and cancer stem cell-like traits in non-small-cell lung carcinoma by activating the Wnt/$\beta$-catenin signaling pathway. On the other extreme, there are also genes which are only identified by BetweenNet. MAGED1 is one such example for breast cancer. Tian et al have shown that BRCA2 suppresses cell proliferation via stabilizing its downstream target MAGED1 [44]. As such, MAGED1 is strongly associated with cancer development by mediating the growth-suppressing function of BRCA2 [45].

## Discussion

Having shown that BetweenNet performs better than existing methods in most of the evaluations, we also investigate the added value of defining outliers based on betweenness centrality. To this end, we replace the outliers of BetweenNet with the outliers found by DriverNet for the same data sets. We observe that BetweenNet performs significantly better than its modified version and DriverNet for all cancer types and for all reference gene sets (Additional file 1: Figures 16-18). These results show that the outlier detection strategy of BetweenNet is critical to its performance.

Lastly, we analyze the running time requirements of the main steps of the BetweenNet algorithm. Computing the betweenness values of all the nodes in an unweighted graph of *n* nodes and *m* edges requires *O*(*nm*) time, since starting from each node a breadth-first search (BFS) is executed until completion to find the shortest path distances necessary for the betweenness values. However since we only consider the shortest paths within a diameter of *k*, the number of edges traversed at each BFS is bounded by $\delta ^{2k+2}$, where $\delta$ denotes the maximum degree of all the nodes in the graph *G* representing the input PPI network. Thus the running time of the betweenness step of BetweenNet is $O(|P||V|\delta ^{2k+2})$. Let *a* denote the average number of outliers per patient and $\mu$ denote the number of genes mutated at least once in the set of samples *P*. The running time of the random-walk step is bounded by $O((|P|a+\mu )^3r)$, where *r* denotes the number of times the *F* matrix is calculated iteratively until convergence. We observe the *a* and $\mu$ values of 870 and 4,335 for lung cancer; 390 and 4,096 for breast cancer; 627 and 11,105 for pan-cancer datasets respectively. We observe that the random walk converges after 3 iterations for all three datasets. For the actual ranking step, the main operations are those of iteratively selecting and removing the maximum rank mutated gene and updating the current ranks of the remaining mutated genes that are also connected to the outliers of the removed gene. Although a more efficient structure such as a priority queue could be employed, since this is not the dominantly time-consuming step of the algorithm we opt for simple node deletions from *B* followed by a linear search for maximum ranking mutated gene. A removed mutated gene is on average incident to $O(\delta a)$ edges in *B*. Thus a single removal of a mutated gene and its neighbors in *B* and the following degree updates costs $O(\delta ^2 a+\mu )$ time and the overall running time of the actual ranking step is $O(\mu (\delta ^2 a+\mu ))$.

## Conclusions

We propose BetweenNet, a novel cancer driver gene prioritization approach that integrates genomic data with the connectivity within PPI networks. One contribution of BetweenNet is the identification of patient specific dysregulated genes with a measure based on betweenness centrality on personalized networks. BetweenNet ranks mutated genes by their effects on dysregulated genes. To characterize these effects, a bipartite influence graph is formed to represent the relations between the mutated genes and dysregulated genes in each patient. Another contribution of BetweenNet is the employment of a random-walk process on the resulting influence bipartite graph. Through careful comparisons, we show that both the use of betweenness centrality metric and the employment of random walk have added values in identification of cancer driver genes. We also demonstrate that BetweenNet outperforms the alternative methods in recovering known reference genes and in providing functionally coherent rankings with three large-scale TCGA datasets: lung cancer, breast cancer, and pan-cancer samples. Additionally, we find that many of our top ranking genes that do not appear in reference cancer gene sets have roles in cancer development based on existing literature. Taken together, our results indicate that BetweenNet effectively integrates genomic data and connectivity information to prioritize cancer driver genes.

## Supplementary Information

**Additional file 1:** Supplementary materials (Supplementary Tables 1–7, Supplementary Figures 1–18).

## Data Availability

The source code and datasets used in this research can be downloaded from https://github.com/abu-compbio/BetweenNET

## Abbreviations

GO: gene ontology
CGC: cancer gene census
TCGA: the cancer genome atlas
PPI: protein-protein interaction
BRCA: breast invasive carcinoma
LUAD: lung adenocarcinoma
LUSC: lung squamous cell carcinoma
LEDA: library of efficient data types and algorithms
AUROC: area under the receiver operating characteristics
GOC: GO consistency
