# Supplementary material for: Ranking cancer drivers via betweenness-based outlier detection and random walks
Source: BMC Bioinformatics. 2021 Feb 10;22:62. doi: 10.1186/s12859-021-03989-w (PMC7877041; doi:10.1186/s12859-021-03989-w)
Supplement: Supplementary file 1 — Additional file 1: Supplementary materials (Supplementary Tables 1–7, Supplementary Figures 1–18). [file 12859_2021_3989_MOESM1_ESM.pdf]

Table 1 Pan-cancer size

Cancer type	Samples
STAD	27
LUSC	16
KIRC	72
KICH	25
KIRP	32
LIHC	49
LUAD	45
BRCA	110
ESCA	11
HNSC	37
THCA	46
COADREAD	25
STES	38
BLCA	15

Table 2 Lung

Datasets	Genes	# of enriched GO terms	# of enriched Reactome pathways	# of enriched KEGG pathways
CGC	723	1353	564	144
CGC (Lung) U NCG (LUNG)	96	-	-	-
CancerMine3	58	-	-	-
CancerMine5	33	-	-	-

Table 3 Breast

Datasets	Genes	# of enriched GO terms	# of enriched Reactome pathways	# of enriched KEGG pathways
CGC	723	1353	564	144
CGC (Breast) U NCG (Breast)	142	-	-	-
CancerMine3	89	-	-	-
CancerMine5	58	-	-	-

Table 4 Pan-cancer

Datasets	Genes	# of enriched GO terms	# of enriched Reactome pathways	# of enriched KEGG pathways
CGC	723	1353	564	144
CancerMine3	169	-	-	-
CancerMine5	120	-	-	-

Table 5 The statistics of top 30 lung cancer driver genes identified by our method

Gene	Rank	CGC	the number of patients with mutations	# of other methods that also rank this gene within the top 30
TP53	1	1	28	4
EGFR	2	1	9	4
TTN	3	0	34	4
LRRK2	4	0	7	4
KEAP1	5	1	11	3
DISC1	6	0	1	2
STK11	7	1	6	2
ATXN1	8	0	2	2
KRAS	9	1	14	3
MET	10	1	6	3
SMAD2	11	1	2	1
PLEC	12	0	4	3
TRAF6	13	0	1	2
NF1	14	1	8	3
RB1	15	1	5	3
IKBKE	16	0	1	1
POT1	17	1	2	2
NLRP12	18	0	6	2
SMAD4	19	1	4	3
RIF1	20	0	4	2
REL	21	1	2	1
ZDHHC17	22	0	2	2
HOXA1	23	0	2	1
FYN	24	0	1	1
APC	25	1	2	1
ANK2	26	0	13	1
MYH9	27	1	5	1
AHNAK	28	0	6	1
IKZF3	29	0	2	1
DYSF	30	0	7	2

Table 6 The statistics of top 30 breast cancer driver genes identified by our method

Gene	Rank	CGC	the number of patients with mutations	# of other methods that also rank this gene within the top 30
TP53	1	1	37	5
TTN	2	0	20	5
MAP3K1	3	1	12	4
PIK3CA	4	1	34	4
GOLGA2	5	0	2	3
FMR1	6	0	2	2
PICK1	7	0	2	3
ERBB2	8	1	2	1
LZTS2	9	0	3	3
MEOX2	10	0	1	2
RIF1	11	0	5	3
ABL1	12	1	3	4
LRRK2	13	0	2	3
ERBB3	14	1	4	4
DISC1	15	0	1	2
PIK3R1	16	1	3	2
HSP90AB1	17	1	1	1
SETDB1	18	1	3	3
IKBKE	19	0	1	2
ATXN1	20	0	1	2
RB1	21	1	3	2
PLCG1	22	1	3	1
YWHAG	23	0	1	0
EWSR1	24	1	2	0
CDH1	25	1	5	2
APP	26	0	2	2
SRGAP2	27	0	4	2
TSG101	28	0	2	1
DLG1	29	0	3	2
MAGED1	30	0	2	0

Table 7 The statistics of top 30 pan cancer driver genes identified by our method

Gene	Rank	CGC	the number of patients with mutations	# of other methods that also rank this gene within the top 30
TP53	1	1	201	5
TTN	2	0	187	5
EGFR	3	1	31	4
VHL	4	1	45	5
LRRK2	5	0	34	4
APC	6	1	45	4
GOLGA2	7	0	13	3
CTNNB1	8	1	32	3
ERBB2	9	1	17	3
HLA-B	10	0	10	1
HTT	11	0	15	2
EP300	12	1	21	3
ATXN1	13	0	11	1
SMAD4	14	1	31	3
ERBB3	15	1	21	3
IKBKE	16	0	7	1
PLEC	17	0	21	2
RB1	18	1	33	3
MAP3K1	19	1	21	2
PIK3R1	20	1	15	1
RIF1	21	0	23	2
PIK3CA	22	1	91	4
HSP90AB1	23	1	4	2
NF1	24	1	38	4
DISC1	25	0	3	1
MCC	26	0	7	1
FMR1	27	0	6	0
TNIK	28	0	10	1
TRAF6	29	0	4	0
CSNK2A1	30	0	9	0

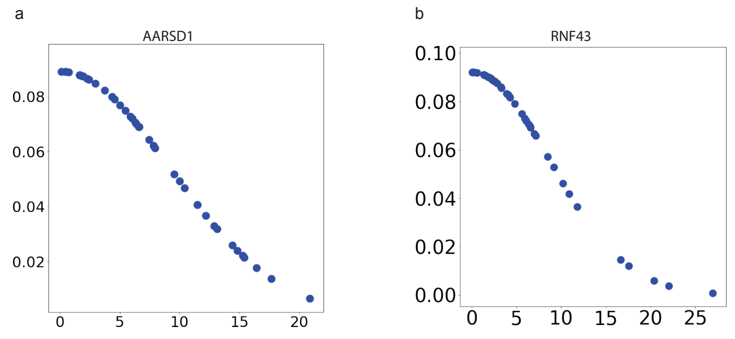

Figure 1 The distribution of betweenness difference values for two selected genes AARSD1 and RNF43.

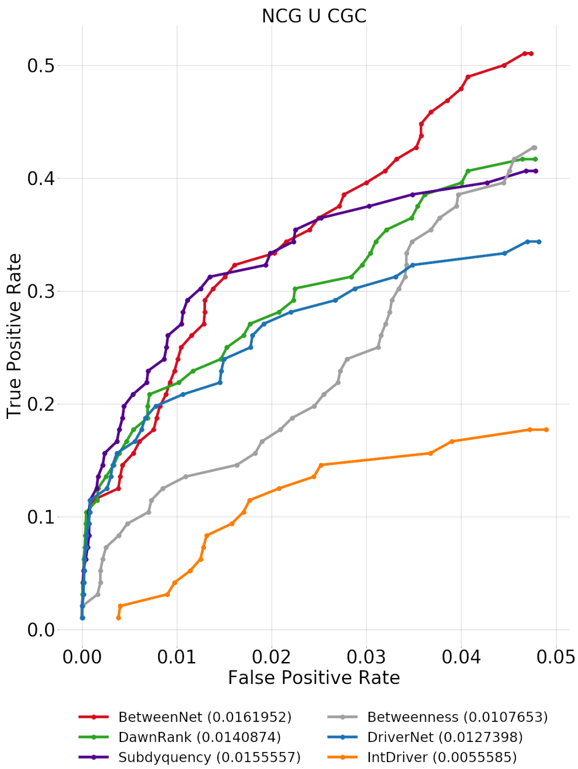

Figure 2 The fraction of recovered reference genes is shown with a ROC curve for lung cancer data where the union of *CGC (Lung)* and *NCG (Lung)* genes are used as reference.

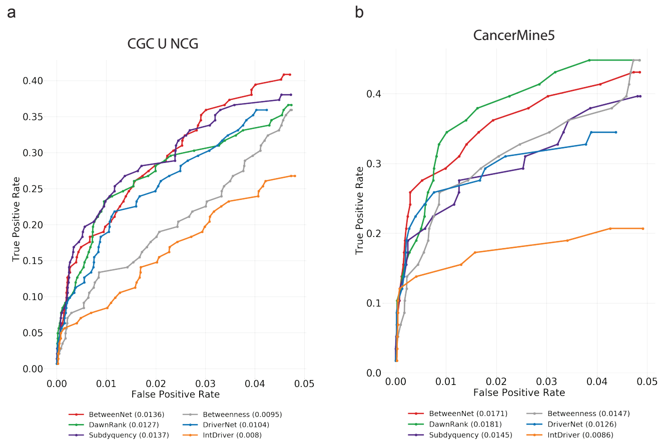

Figure 3 The fraction of recovered reference genes is shown with a ROC curve for breast cancer data where a) the union of *CGC (Breast)* and *NCG (Breast)*, b) *CancerMine5* genes are used as reference.

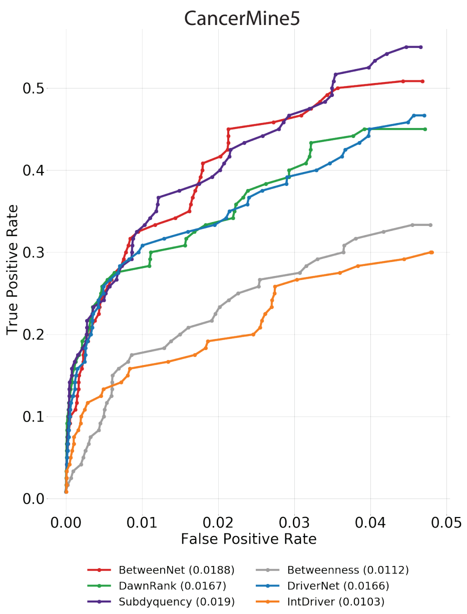

Figure 4 The fraction of recovered reference genes is shown with a ROC curve for pan-cancer data where *CancerMine5* genes are used as reference.

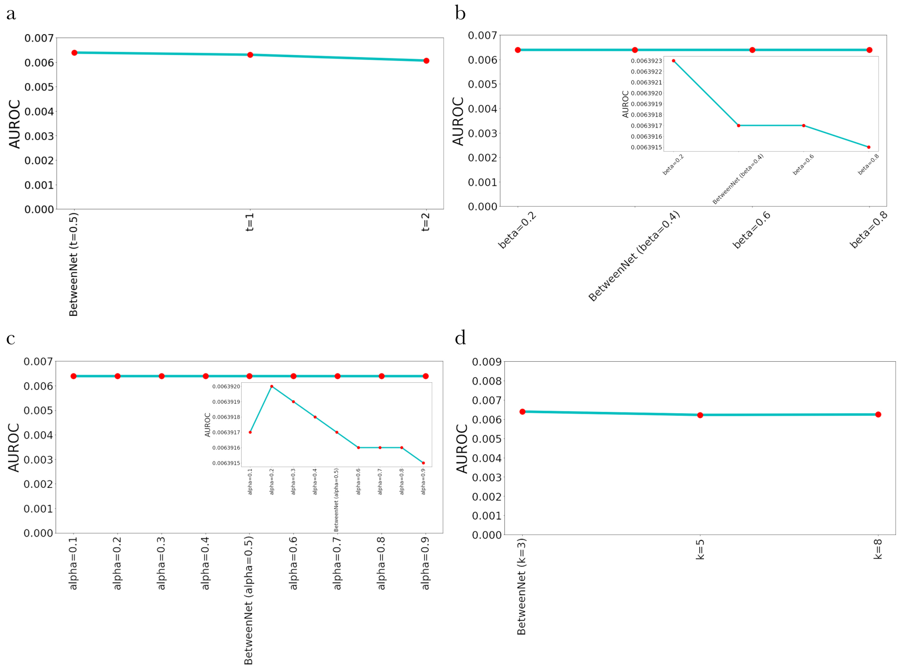

Figure 5 Sensitivity test of parameters of BetweenNet on lung cancer data when *CGC* is used as reference. a) AUROC values with different thresholds for defining outlier genes b) AUROC values with different restart probability values (β) c) AUROC values with different alpha values d) AUROC values with different k values.

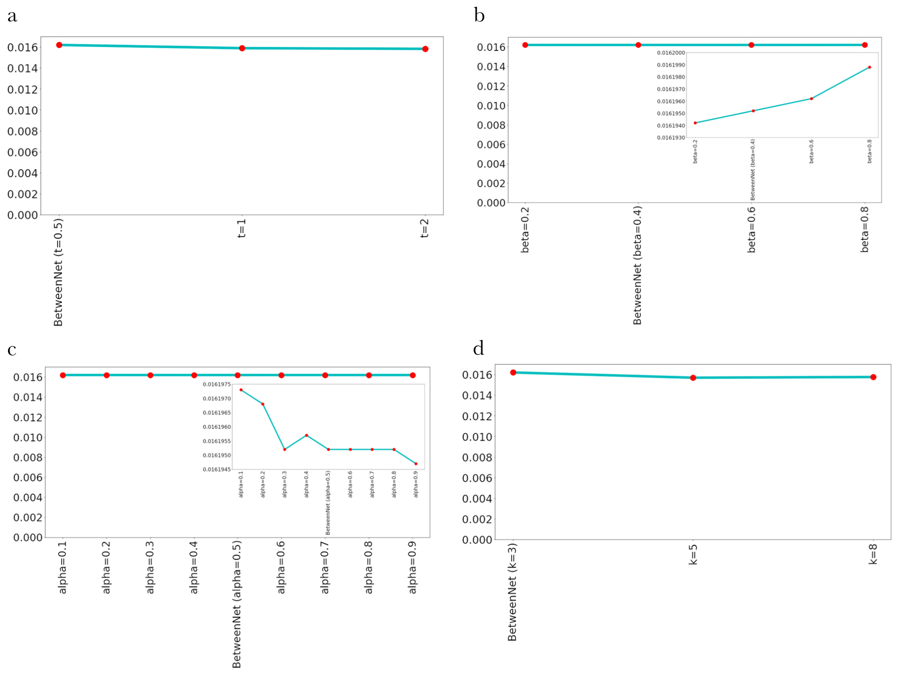

Figure 6 Sensitivity test of parameters of BetweenNet on lung cancer data when the union of *CGC (Lung)* and *NCG (Lung)* is used as reference. a) AUROC values with different thresholds for defining outlier genes b) AUROC values with different restart probability values (β) c) AUROC values with different alpha values d) AUROC values with different k values.

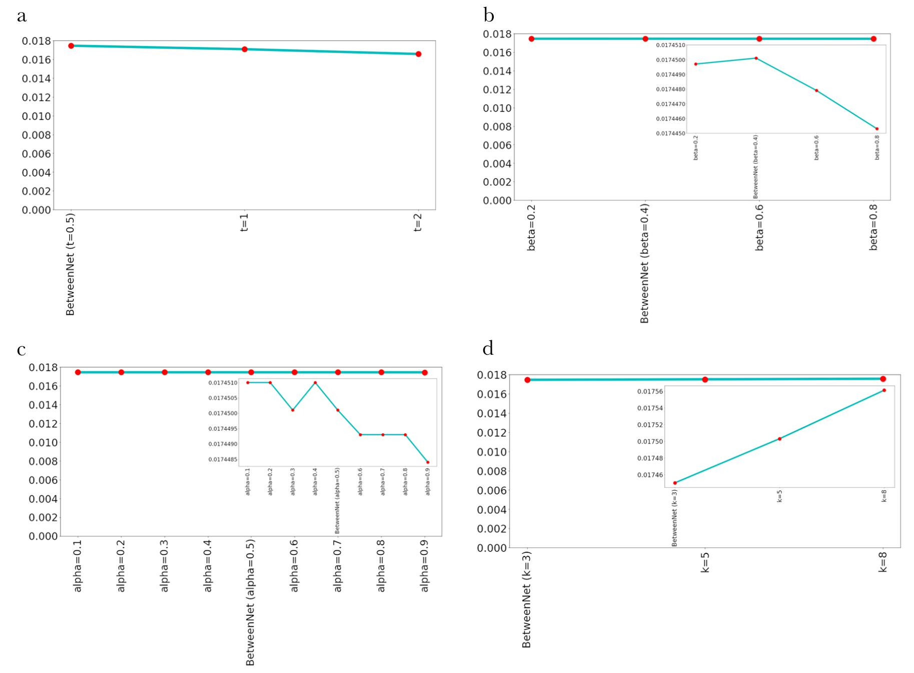

Figure 7 Sensitivity test of parameters of BetweenNet on lung cancer data when *CancerMine3* is used as reference. a) AUROC values with different thresholds for defining outlier genes b) AUROC values with different restart probability values (β) c) AUROC values with different alpha values d) AUROC values with different k values.

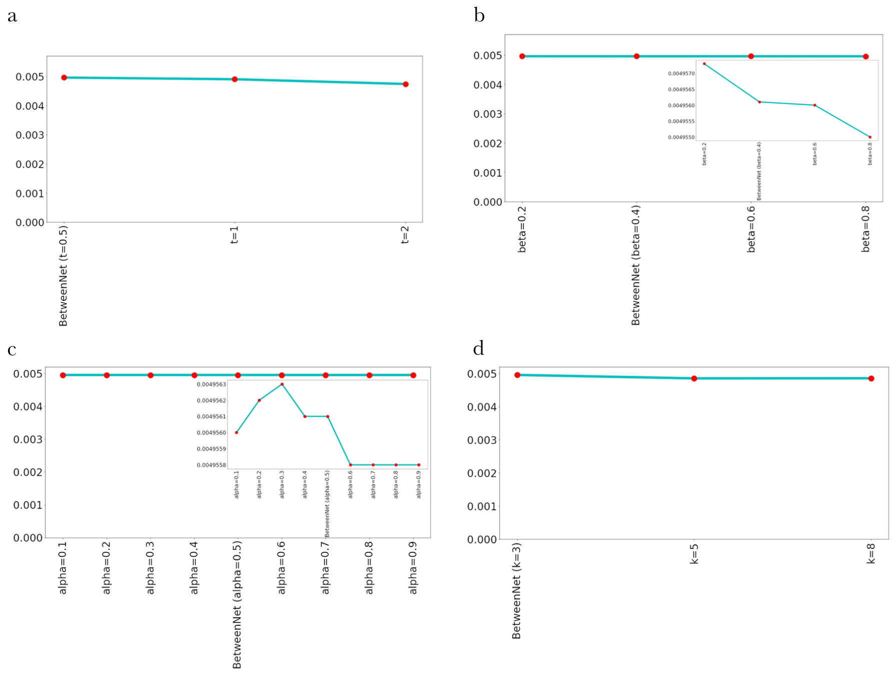

Figure 8 Sensitivity test of parameters of BetweenNet on lung cancer data when *CGC* rare genes are used as reference. a) AUROC values with different thresholds for defining outlier genes b) AUROC values with different restart probability values (β) c) AUROC values with different alpha values d) AUROC values with different k values.

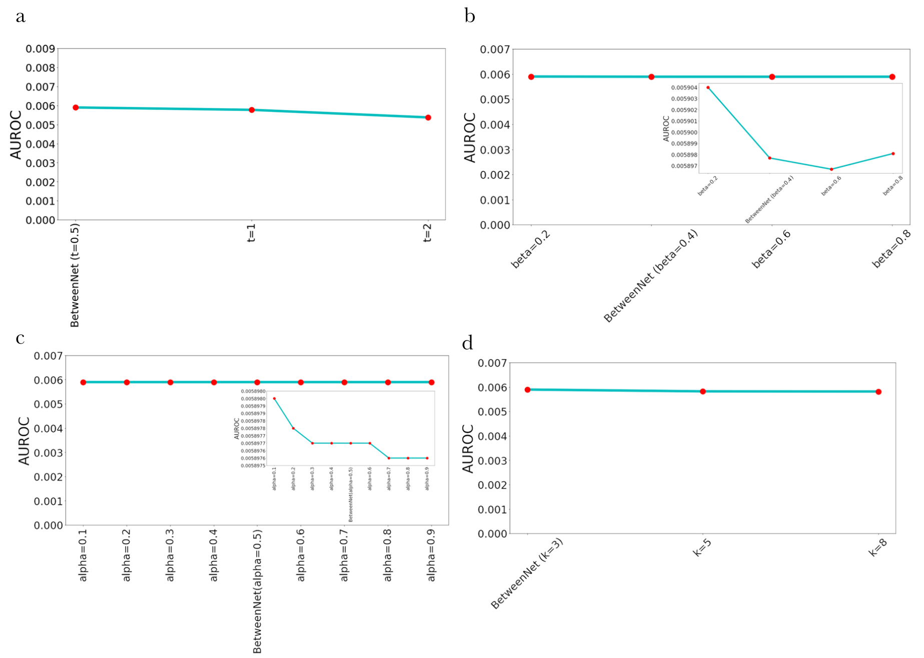

Figure 9 Sensitivity test of parameters of BetweenNet on breast cancer data when *CGC* is used as reference. a) AUROC values with different thresholds for defining outlier genes b) AUROC values with different restart probability values (β) c) AUROC values with different alpha values d) AUROC values with different k values.

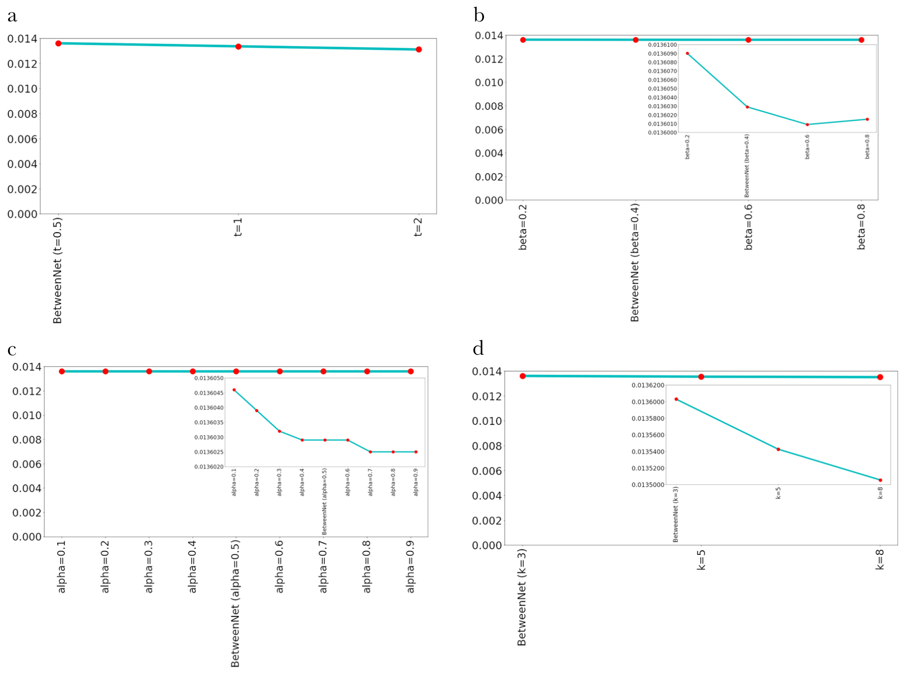

Figure 10 Sensitivity test of parameters of BetweenNet on breast cancer data when the union of *CGC (Breast)* and *NCG (Breast)* is used as reference. a) AUROC values with different thresholds for defining outlier genes b) AUROC values with different restart probability values (β) c) AUROC values with different alpha values d) AUROC values with different k values.

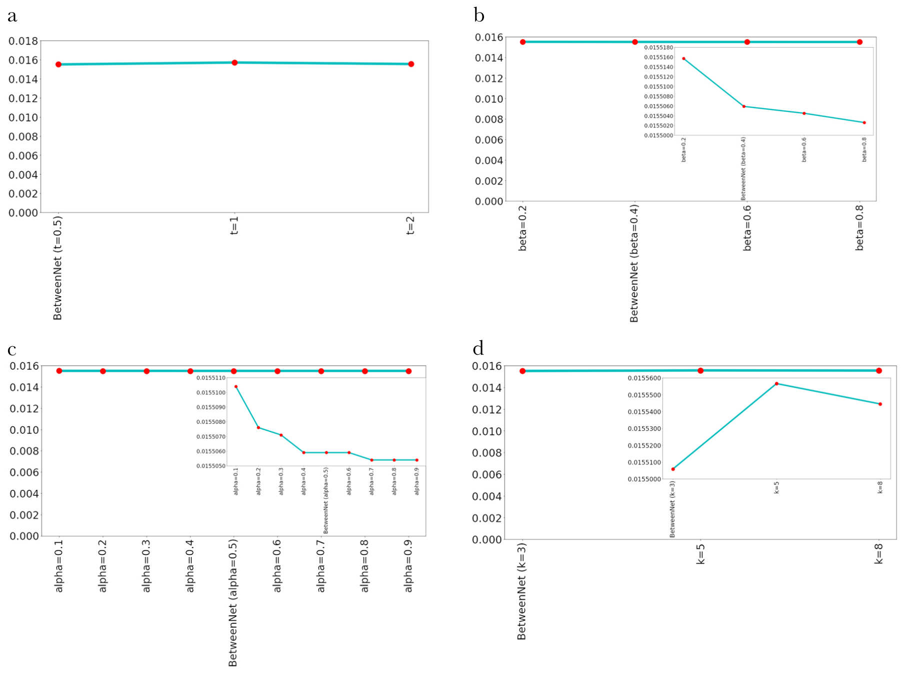

Figure 11 Sensitivity test of parameters of BetweenNet on breast cancer data when *CancerMine3* is used as reference. a) AUROC values with different thresholds for defining outlier genes b) AUROC values with different restart probability values (β) c) AUROC values with different alpha values d) AUROC values with different k values.

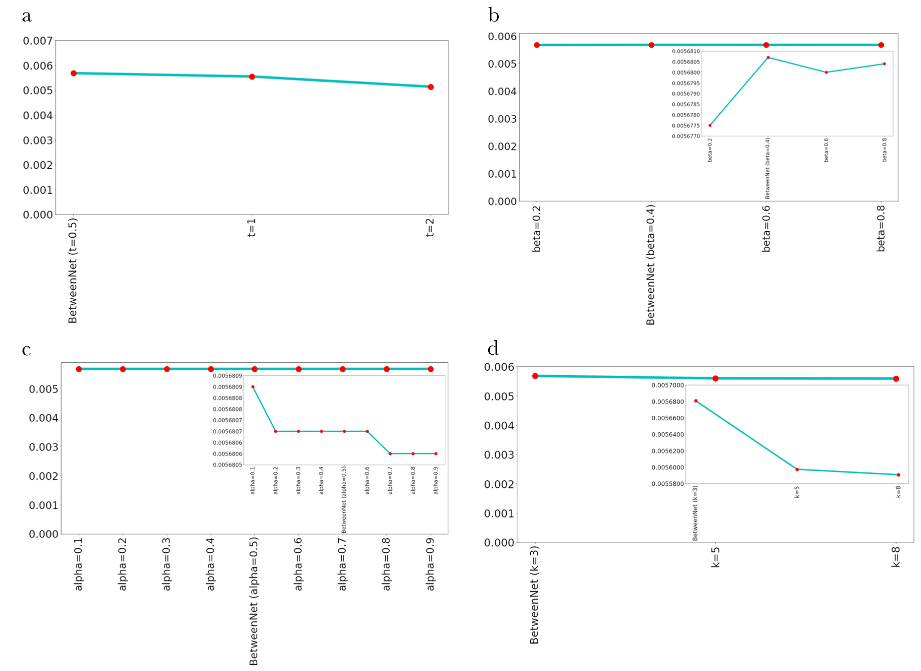

Figure 12 Sensitivity test of parameters of BetweenNet on breast cancer data when *CGC* rare genes are used as reference. a) AUROC values with different thresholds for defining outlier genes b) AUROC values with different restart probability values (β) c) AUROC values with different alpha values d) AUROC values with different k values.

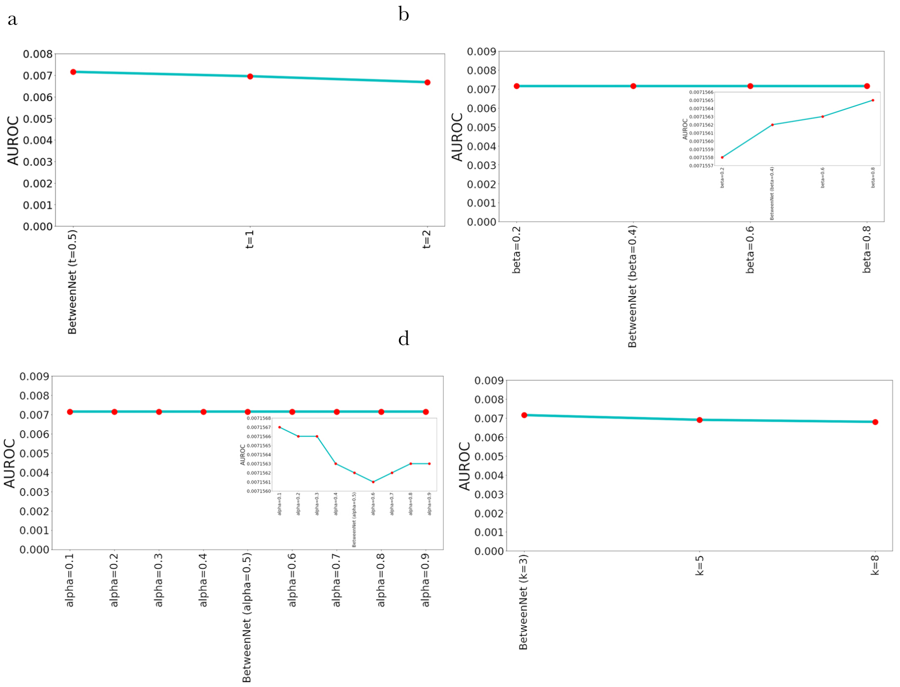

Figure 13 Sensitivity test of parameters of BetweenNet on pan cancer data when *CGC* is used as reference. a) AUROC values with different thresholds for defining outlier genes b) AUROC values with different restart probability values (β) c) AUROC values with different alpha values d) AUROC values with different k values.

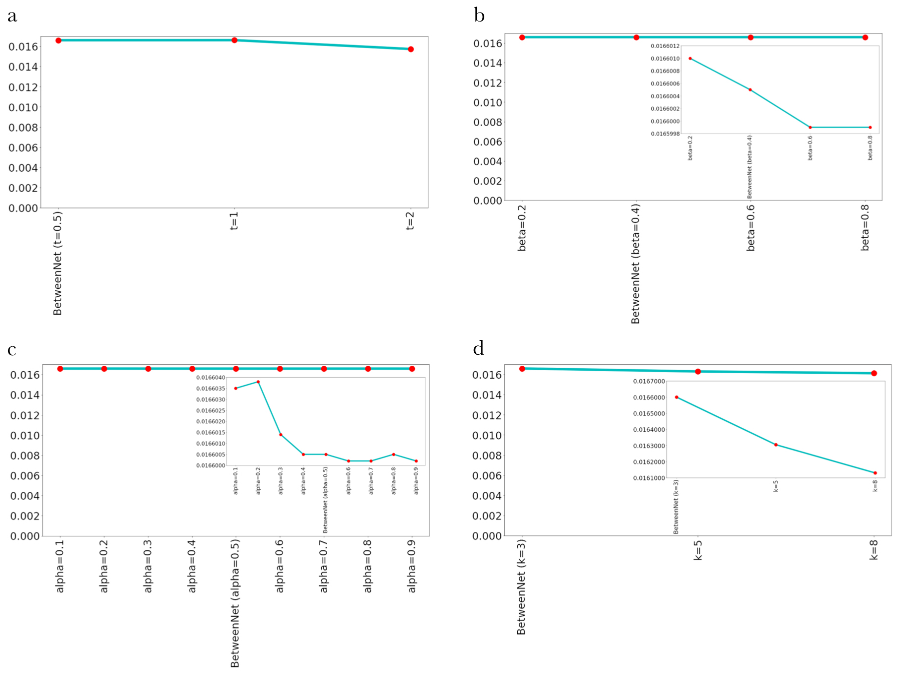

Figure 14 Sensitivity test of parameters of BetweenNet on pan cancer data when *CancerMine3* is used as reference. a) AUROC values with different thresholds for defining outlier genes b) AUROC values with different restart probability values (β) c) AUROC values with different alpha values d) AUROC values with different k values.

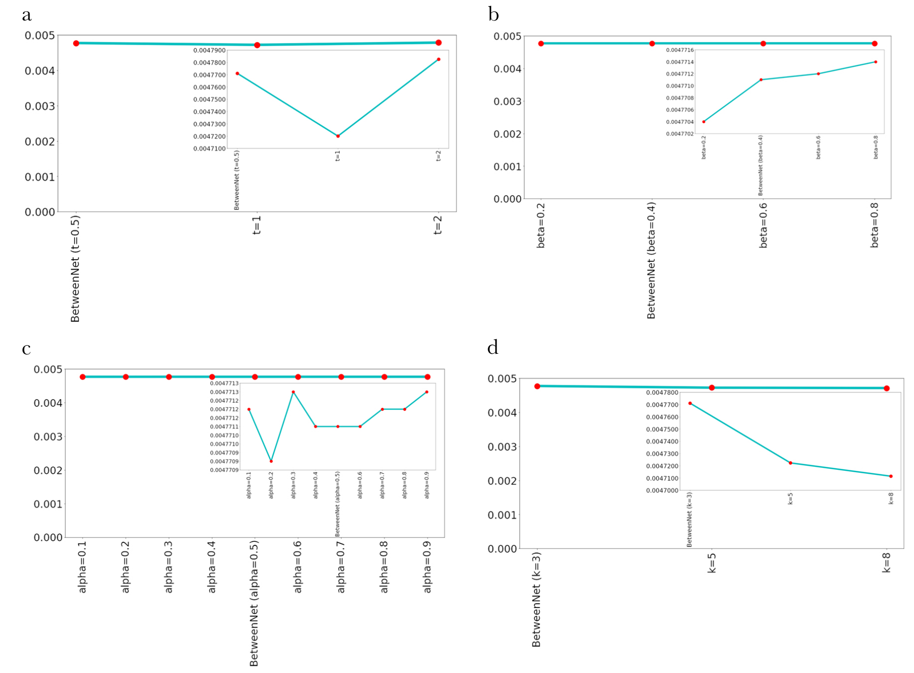

Figure 15 Sensitivity test of parameters of BetweenNet on pan cancer data when *CGC* rare genes are used as reference. a) AUROC values with different thresholds for defining outlier genes b) AUROC values with different restart probability values (β) c) AUROC values with different alpha values d) AUROC values with different k values.

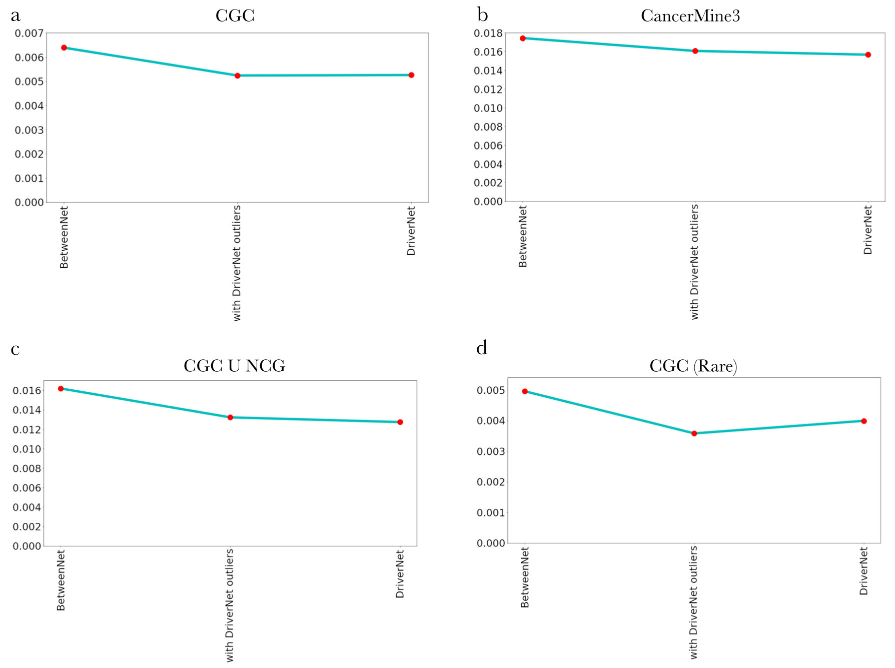

Figure 16 AUROC values of BetweenNet, DriverNet and a modified version of BetweenNet where outliers are defined according to DriverNet's outlier definition method. where a) *CGC* b) *CancerMine3* c) the union of *CGC (Lung)* and *NCG (Lung)*, d) *CGC rare* genes are used as reference.

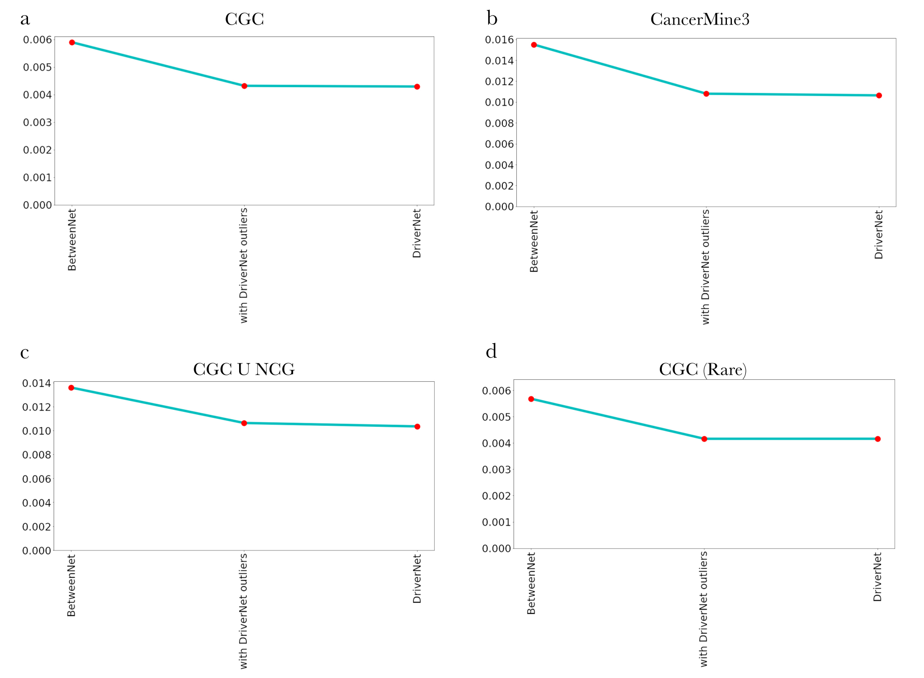

Figure 17 AUROC values of BetweenNet, DriverNet and a modified version of BetweenNet where outliers are defined according to DriverNet's outlier definition method. where a) *CGC* b) *CancerMine3* c) the union of *CGC (Breast)* and *NCG (Breast)*, d) *CGC rare* genes are used as reference.

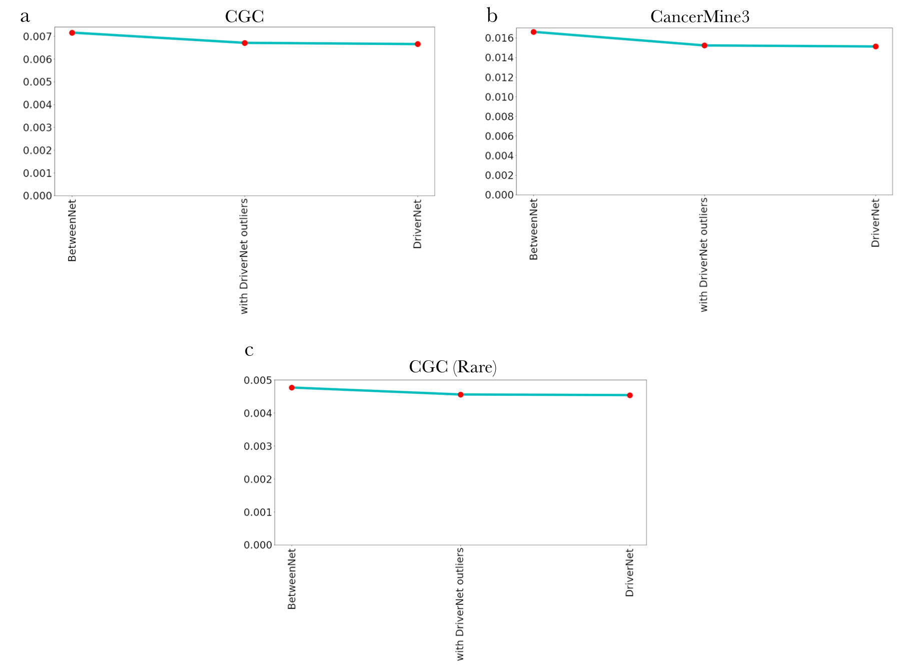

Figure 18 AUROC values of BetweenNet, DriverNet and a modified version of BetweenNet where outliers are defined according to DriverNet's outlier definition method. where a) *CGC* b) *CancerMine3* and c) *CGC rare* genes are used as reference.
